# Sequencing of complete mitochondrial genome for Tsinling Tree Toad (*Hyla tsinlingensis*)

**DOI:** 10.1080/23802359.2016.1186515

**Published:** 2016-07-08

**Authors:** Xing Kang, Zhonglou Sun, Weibo Guo, Jun Wu, Lifu Qian, Tao Pan, Hui Wang, Kai Li, Baowei Zhang

**Affiliations:** a School of Life Sciences, Anhui University, Hefei, Anhui, China;; b Ministry of Environmental Protection, Nanjing Institute of Environmental Sciences, Nanjing, Jiangsu, China

**Keywords:** *Hyla tsinlingensis*, mitochondrial genome, phyolgentic tree

## Abstract

The complete mitochondrial genome sequence of *Hyla tsinlingensis* was determined in this research. The length of mitogenome is 17850 bp, including 13 protein-coding genes, 22 tRNA genes, 2 rRNA genes, 1 O_L_ and 1 control region. The phylogentic tree was reconstructed using the BI method based on concatenated nucleotide sequences of mtDNA genes (12S ribosomal small subunit gene/12S rRNA; NADH dehydrogenase subunit 1 gene/ND1, including adjacent transfer RNAs and the partial 16S ribosomal large subunit gene). The phylogenetic tree was split into two clades, Clade A and Clade B. The *H. tsinlingensis* which we determined clustered into Clade A.

The Tsinling Tree Toad, *Hyla tsinlingensis* is a kind of small and semiaquatic vertebrate, belonging to the genus *Hyla* of family Hylidae. This species is endemic to China, with a restricted and patchy distribution in Tsinling and Dabie Mountains (Hu et al. 1966; Zhang et al. 2016). Widely inhabit in paddy fields or edges of rivers, for their reproduction, the *H. tsinlingensis* prefers to open waters. Taking habitat is shrinking into consideration (Zhang et al. 2016), it is extremely urgent to take measure to protect *H. tsinlingensis*.

The *H. tsinlingensis* sample was collected in Yaoluoping nature reserve, Anqing of Anhui Province, China (N 31°1′15.12″, E 116°8′39.32″) in April 2014. Presently, the specimen was stored in the Key Laboratory of Eco-engineering and Bio-technique, School of Life Sciences, Anhui University. We clipped muscle tissue to extract the whole genomic DNA using a standard proteinase-K/phenol–chloroform protocol (Sambrook & Russell 2006). The entire mitogenome was amplified using 19 pairs of primers by polymerase chain reaction (PCR). Here, we sequenced the complete mitochondrial genome of *H. tsinlingensis* and submitted it to GenBank (accession no. KU601448).

The complete mitogenome sequence of *H. tsinlingensis* is 17,850 bp in length, and the gene order was identical to *Hyla chinensis* (Zhang et al. 2005), including 13 protein-coding genes, 22 tRNA genes, 2 rRNA genes, 1 O_L_ and 1 control region. The base composition of the mitogenome was 30.1% A, 24.9% C, 14.6% G and 30.4% T. The *ND6* subunit gene, rep-origin and eight tRNA genes (*tRNA^Pro^*, *tRNA^Gln,^ tRNA^Ala^*, *tRNA^Asn^*, *tRNA^Cys^*, *tRNA^Tyr^*, *tRNA^Ser^*^(UCN)^, and *tRNA^Glu^*) were encoded on the L-strand, the remaining genes were encoded on the H-strand.

The phylogenetic tree were reconstructed by Bayesian inference (BI) with MrBayes 3.2.2 (Ronquist & Huelsenbeck 2003) methods based on concatenated nucleotide sequences of mtDNA genes (12S ribosomal small subunit gene/12S rRNA; NADH dehydrogenase subunit 1 gene/ND1, including adjacent transfer RNAs and the partial 16S ribosomal large subunit gene). *Bufo tibetanus*, *B. gargarizans*, *Rana sylvatica*, and *R. dybowskii* were selected as outgroups. In Bayesian process, the optimal substitution model (12S: GTR + G; ND1: HKY + I + G) was implemented via JModelTest 2 (Darriba et al. 2012). Two parallel runs of Markov Chain Monte Carlo (MCMC) were analyzed for 1,000,000 generations, sampling every 1000 generations and discarded 1000 trees as burn-in. As shown in Figure 1, Bayesian analysis suggested the phylogenetic tree was split into two well-supported major clades, Clade A and Clade B. The *H. tsinlingensis* which we determined the mitochondrial genome (the blue arrow) was clustered into Clade A. Huang et al. (2015) also researched the complete mtDNA of *H. tsinlingensis*, however, this species (the red arrow) was clustered into Clade B in Figure 1. Moreover, this species was deeply nested within another two species of *Hyla immaculata*, so we suspected that the species in Huang et al. (2015) can been recognized as the *H. immaculate sp.*

**Figure 1.**
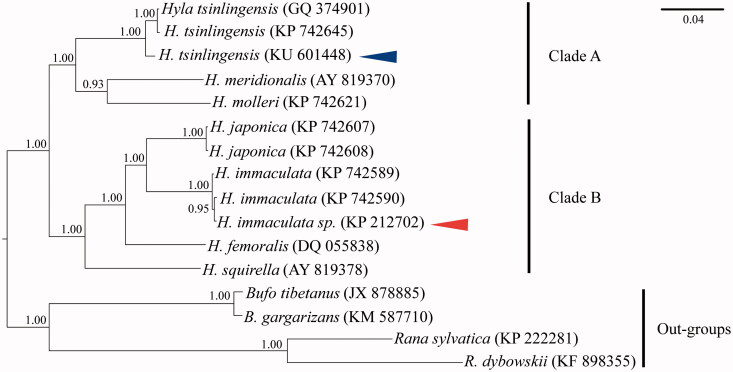
The phylogenetic tree inferred by the BI method, based on 12S and ND1 genes. Numbers at the nodes are bootstrap values of BI method. The GenBank accession number of 12S are listed next to the species’ names, and the number of ND1 are *Hyla tsinlingensis* (GQ 374905), *H. tsinlingensis* (KP 742764), *H. tsinlingensis* (KU 601448), *H. meridionalis* (AY 819502), *H. molleri* (KP 742743), *H. japonica* (KP 742734), *H. japonica* (KP 742735), *H. immaculata* (KP 742717), *H. immaculata* (KP 742718), *H. immaculata* sp. (KP 212702), *H. femoralis* (DQ 055819), *H. squirella* (AY 819510), *Bufo tibetanus* (JX 878885), *B. gargarizans* (KM 587710), *Rana sylvatica* (KP 222281), *R. dybowskii* (KF 898355). *H. tsinlingensis* position is indicated in blue arrow, *H. immaculata* sp. position is indicated in red arrow.

Mitochondrial DNA plays a significant role in the phylogeney. We expect to provide a useful database for analyzing the phylogenetic relationship within *H. tsinlingensis* via this research.
